# Development and validation of a simple-to-use clinical nomogram for predicting obstructive sleep apnea

**DOI:** 10.1186/s12890-019-0782-1

**Published:** 2019-01-18

**Authors:** Huajun Xu, Xiaolong Zhao, Yue Shi, Xinyi Li, Yingjun Qian, Jianyin Zou, Hongliang Yi, Hengye Huang, Jian Guan, Shankai Yin

**Affiliations:** 1 Department of Otolaryngology Head and Neck Surgery & Center of Sleep Medicine, Shanghai Jiao Tong University Affiliated Sixth People’s Hospital, Yishan Road 600, Shanghai, 200233 China; 2 Otolaryngological, Institute of Shanghai Jiao Tong University, Yishan Road 600, Shanghai, 200233 China; 3 Shanghai Key Laboratory of Sleep Disordered Breathing, Shanghai Jiao Tong University Affiliated Sixth People’s Hospital, 600 Yishan Road, Shanghai, 200233 China; 4 Department of Epidemiology, School of Public Health, Shanghai Jiao Tong University, 225South Chongqing Road, Shanghai, 200020 China

**Keywords:** Obstructive sleep apnea, Nomogram, Risk factor

## Abstract

**Background:**

The high cost and low availability of polysomnography (PSG) limits the timely diagnosis of OSA. Herein, we developed and validated a simple-to-use nomogram for predicting OSA.

**Methods:**

We collected and analyzed the cross-sectional data of 4162 participants with suspected OSA, seen at our sleep center between 2007 and 2016. Demographic, biochemical and anthropometric data, as well as sleep parameters were obtained. A least absolute shrinkage and selection operator (LASSO) regression model was used to reduce data dimensionality, select factors, and construct the nomogram. The performance of the nomogram was assessed using calibration and discrimination. Internal validation was also performed.

**Results:**

The LASSO regression analysis identified age, sex, body mass index, neck circumference, waist circumference, glucose, insulin, and apolipoprotein B as significant predictive factors of OSA. Our nomogram model showed good discrimination and calibration in terms of predicting OSA, and had a C-index value of 0.839 according to the internal validation. Discrimination and calibration in the validation group was also good (C-index = 0.820). The nomogram identified individuals at risk for OSA with an area under the curve (AUC) of 0.84 [95% confidence interval (CI), 0.83–0.86].

**Conclusions:**

Our simple-to-use nomogram is not intended to replace standard PSG, but will help physicians better make decisions on PSG arrangement for the patients referred to sleep center.

**Electronic supplementary material:**

The online version of this article (10.1186/s12890-019-0782-1) contains supplementary material, which is available to authorized users.

## Background

Obstructive sleep apnea (OSA) is one of the most common types of sleep-disordered breathing. One notable characteristic of OSA is recurrent episodes of partial/complete pharyngeal collapse and subsequently reduced oronasal airflow, or even cessation of breathing, during sleep [1]. OSA is closely associated with impairments in daily activities and social functioning due to daytime sleepiness and fatigue, in turn caused by sleep fragmentation and clinical sequelae such as hypertension, diabetes, dyslipidemia, cognitive dysfunction, cardiovascular events and even all-cause mortality [2–7]. The prevalence of OSA is high and has increased with the obesity epidemic according to data gathered since the 1990s [8–10]; however, most cases remain undiagnosed and thus the disease is undertreated, resulting in a high social and economic burden.

Although awareness of the prevalence of OSA and its clinical sequelae have increased recently based on data from western countries, the wait time for polysomnography (PSG) ranges from 2 to 60 months [11]; thus, limited access to PSG, which also involved significant expense, remains a major issue. Chinese patients with OSA also need time to reach the aforementioned treatment plan due to a lack of readily available PSG labs. A delayed diagnosis in turn results in delayed OSA treatment and, consequently, more comorbidities. Thus, a simple-to-use and reliable method to identify and triage patients at high risk for OSA is urgently needed.

Well-designed questionnaires [i.e., the STOP-Bang questionnaire (SBQ), STOP questionnaire (STOP), Epworth Sleepiness Scale (ESS) and the Berlin questionnaire (BQ)] have been designed as substitute methods for diagnosing OSA in the absence of standard PSG. However, the results have been unsatisfactory. In a recent meta-analysis, pooled specificity was low, ranging from 42 to 65% [12]. Furthermore, questionnaires can be susceptible to bias and snoring is not amenable to self-report, as OSA symptoms occur during sleep. Uncertainty exists about the accuracy and clinical utility of the above-mentioned screening tools [13]. An optimized diagnostic tool that combines multiple, objective clinical biomarkers, to avoid bias, has yet to be developed.

No study has determined whether using a combination of objective, clinical biomarkers confers a good diagnostic ability for OSA. Therefore, the aim of our study was to develop and validate a simple-to-use nomogram that incorporated objective demographic, biochemical, and anthropometric parameters to determine whether this nomogram minimized the number of missed OSA diagnoses.

## Methods

Our study used a cross-sectional design, was performed in accordance with Declaration of Helsinki and its amendments, and was approved by the Ethics Committee of Shanghai Jiaotong University Affiliated Sixth People’s Hospital, Shanghai, China. Written informed consent was obtained from each subject before study enrollment.

### Study population

Subjects who complained of snoring or other symptoms (such as daytime sleepiness) of OSA, and who were referred to our sleep center between 2007 and 2016 were consecutively enrolled. All subjects completed questionnaires, which were collected and checked by two independent investigators and captured medical history and health status of the subjects. The exclusion criteria were age less than 18 years; serious systemic disease (i.e., congestive heart failure, severe intrinsic pulmonary disease, chronic kidney disease or hepatic disease); pregnancy; had anti-diabetes and taken lowing-lipids drugs and previous OSA treatment. Subjects with missing clinical data were also excluded from the final analysis on agreement among all authors.

### Data collection and analysis

#### Anthropometric measurements

Height and weight were measured with the subjects in light clothing and bare feet, as previously described [14]. Weight was measured by an electronic scale. Height was measured from the feet to the head, as the maximum distance when subjects were standing up straight. Body mass index (BMI) was defined as weight (kg) divided by height in meters^2^. Neck circumference (NC) was measured at the level of the cricothyroid membrane; hip circumference (HC) was measured as the maximum girth at the greater trochanters; waist circumference (WC) was measured at the middle of the lower costal margin and iliac crest. All anthropometric data mentioned-above were recorded twice.

#### Biochemical measurements

A fasting blood sample was drawn from the vein of each subject at 7 AM after PSG monitoring. The serum lipid profile, including total cholesterol (TC), triglycerides (TG), high-density lipoprotein cholesterol (HDL-C), low-density lipoprotein cholesterol (LDL-C), apolipoprotein A-I (ApoA-I), apolipoprotein B (ApoB), apolipoprotein E, and lipoprotein (a) [Lp(a)] were measured using routine procedures at our hospital laboratory, as previously described [14]. Serum glucose levels were measured using an auto analyzer (Hitachi, Tokyo, Japan), and serum insulin levels were measured using an immunoradiological method [15].

#### Sleep evaluation

Objective sleepiness was evaluated by standard PSG (Alice 4 or 5; Respironics, Pittsburgh, PA, USA) according to the American Academy of Sleep Medicine (AASM) 2007 guidelines [16]. A posture and snoring sensor was equipped and an electroencephalogram, bilateral electrooculogram, modified lead II electrocardiogram, bipolar chin electromyogram, oral airflow, nasal airflow (with nasal cannula), pulse oximetry, thoracic and abdominal respiratory effort were obtained. The sleep recordings were staged automatically and then checked by a skilled technician manually.

Apnea was defined as an absence of oronasal airflow by at-least 90% relative to baseline and lasting ≥10 s. Hypopnea was defined as any upper airflow reduction of 50% for at least 10 s, accompanied by either a decrease in oxyhemoglobin saturation at least 3% or terminated by awakening. Arousal was defined as abrupt shifts in EEG electroencephalographic frequency lasting for ≥3 s [16]. The apnea-hypopnea index (AHI) was given by the number of apnea and hypopnea events per hour of sleep. The oxygen desaturation index was defined as the total number of episodes of oxyhemoglobin desaturation ≥3% per hour of sleep. The micro-arousal index was defined as the average number of arousals per hour of sleep. OSAS was diagnosed as the AHI ≥ 5 times per hour. OSAS was classified as mild (5~ 15), moderate (15 ~ 30), or severe (≥ 30), respectively [16].

#### Statistical methodology

The statistical analysis was conducted using R (ver. 3.0.1, Vienna, Austria) and MedCalc software (ver. 12.7.3, Ostend, Belgium). The glmnet package in R was used for the least absolute shrinkage and selection operator (LASSO) logistic regression, and significant factors there in were used to construct the nomogram [17], for detail, please see Additional file 1. Validation was conducted using one thousand bootstrap analyses. Calibration diagrams were established as previously described [18]. To evaluate the predictive or discriminatory abilities of this model, an index of probability of concordance (C-index) was calculated among predicted and actual outcomes [19]. The C-index had a range from 0.5 to 1.0, with 0.5 was considered to be random chance; 1.0 was denoted as perfect discrimination [19]. The LASSO feature regression model was used to distinguish OSA from non-OSA cases. Area under the curve (AUC) in receiver operating characteristic (ROC) analysis was used to evaluate predictive accuracy. Two-sided *p*-values < 0.05 was considered as statistical significance.

## Results

### Demographic characteristics of the study subjects

A total of 4162 consecutive participants who underwent full-night standard PSG were finally included in our study. We randomly selected 2913 subjects (70%) for inclusion in the training group, and the remaining 1249 (30%) were assigned to the validation group using a random number table. The demographic characteristics of the training and validation groups are summarized in Table 1. No obvious differences were found between the two OSA groups or two non-OSA groups in terms of demographic characteristics. Patients in both OSA groups were older, more obese, and had poorer metabolic profiles and sleep variables, aside from ApoA-I and Lp(a) in the validation group (Table 1).

**Table 1:** Clinical characteristics of the training and validation group

Characteristics	Training group (*n* = 2913)			Validation group (*n* = 1249)		
Demographics	non-OSA (*n* = 537)	OSA (*n* = 2376)	*P* value	non-OSA (*n* = 238)	OSA (*n* = 1011)	*P* value
Age, years	37(30~47)	42(34~53)	< 0.001	37(30~47)	43(34~53)	< 0.001
Male (%)	58.8	84.7	< 0.001	61.8	84.6	< 0.001
BMI, Kg/m^2^	23.71(21.79~25.52)	27.04(24.77~29.39)	< 0.001	23.28(21.29~25.71)	26.45(24.45~29.06)	< 0.001
NC, cm	36(34~39)	40(38~42)	< 0.001	36.75(34~38)	40(38~42)	< 0.001
WC, cm	86(80~92)	97(91~103)	< 0.001	85 (79~92)	96 (90~103)	< 0.001
HC, cm	96(92~100)	102(97~106)	< 0.001	96(92~100)	101(97~106)	< 0.001
Glucose (mmol/L)	5.01(4.65~5.31)	5.31(4.97~5.82)	< 0.001	4.97(4.65~5.30)	5.28(4.94~5.77)	< 0.001
Insulin (uU/ml)	7.35(5.23~10.49)	11.65(7.96~16.90)	< 0.001	7.11(4.92~9.83)	10.18(6.75~15.78)	< 0.001
SBP, mmHg	120(110~126)	125(117~135)	< 0.001	120(111~126)	125(116~135)	< 0.001
DBP, mmHg	77(70~81)	80(74~88)	< 0.001	77(70~82)	80(73~87)	< 0.001
TC	4.36(3.76~4.91)	4.75(4.20~5.35)	< 0.001	4.30(3.75~4.95)	4.78(4.19~5.40)	< 0.001
TG	1.10(0.75~1.58)	1.63(1.16~2.38)	< 0.001	1.12(0.72~1.62)	1.62(1.12~2.35)	< 0.001
HDL-C	1.10(0.96~1.285)	1.02(0.90~1.18)	< 0.001	1.10(0.96~1.29)	1.03(0.90~1.18)	< 0.001
LDL-C	2.63(2.14~3.14)	3.02(2.53~3.53)	< 0.001	2.63(2.18~3.23)	3.02(2.50~3.59)	< 0.001
ApoA-I	1.10(0.96~1.25)	1.05(0.95~1.19)	< 0.001	1.07(0.96~1.22)	1.07(0.96~1.19)	0.409
ApoB	0.74(0.63~0.86)	0.84(0.74~0.96)	< 0.001	0.72(0.63~0.87)	0.84(0.74~0.96)	< 0.001
ApoE	3.89(3.22~4.69)	4.32(3.55~5.46)	< 0.001	3.81(3.10~4.78)	4.34(3.58~5.38)	< 0.001
Lpa	7.88(4.29~16.85)	7.4(3.8~15.18)	0.037	7.70(4.25~16.35)	7.7(3.8~15.3)	0.532
AHI	1.4(0.5~2.9)	40.9(18.7~62.1)	< 0.001	1.85(0.60~3.40)	38.9(17.8~61.2)	< 0.001
Mean SaO_2_	97(96~97)	94(92~95.8)	< 0.001	97(96~97.45)	94(92~95.7)	< 0.001
Minimum SaO_2_	94(91~96)	78(68~85)	< 0.001	94(91~96)	79(68~85)	< 0.001
ODI	1.6 (0.6~3.5)	41.2(18.13~63.5)	< 0.001	1.8(0.7~3.5)	38.2(18.2~63)	< 0.001
MAI	11.8(7.6~19.1)	25(13.2~43.78)	< 0.001	12.8(8.05~20.43)	25.7(13.8~45.2)	< 0.001

The data are presented as means and standard deviation; skewed data are presented as the median (IQR), and categorical data as the number (percentage)

Differences in the baseline characteristics among the two groups were examined using independent samples t test or Mann–Whitney *U* test for continuous variables and chi-square test or Fisher’s exact test for categorical variables

*OSA* obstructive sleep apnea, *BMI* body mass index, *NC* neck circumference, *WC* waist circumference, *HC* hip circumference, *SBP* systolic blood pressure, *DBP* diastolic blood pressure, *TC* total cholesterol, *TG* triglyceride, *HDL-C* high-density lipoprotein cholesterol, *LDL-C* low-density lipoprotein; cholesterol, *ApoA-I* apolipoprotein A-I, *ApoB* apolipoprotein B, *AHI* apnoea–hypopnea index, *SaO*_*2*_ oxygen saturation, *ODI* oxygen desaturation index, *MAI* micro-arousal index

### Factor selection for the predictive model

**Fig. 1:**
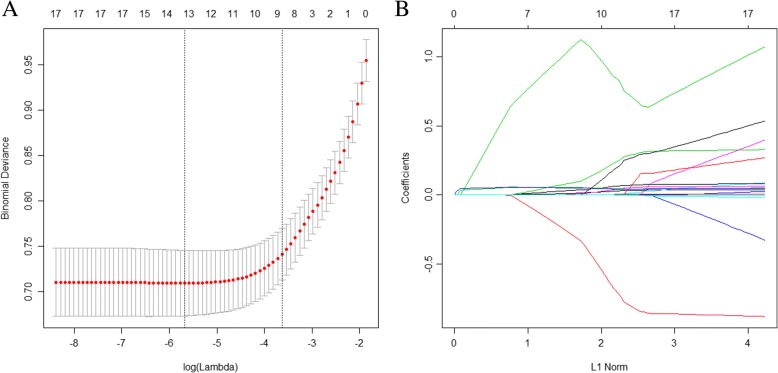
Factor selection using the LASSO logistic regression model. **a** LASSO coefficients of 18 candidate variables. **b** Identification of the optimal penalization coefficient (λ) in the LASSO model was achieved by 10-fold cross-validation and the minimum criterion. The left vertical line represents the minimum error, and the right vertical line represents the cross-validated error within 1 standard error of the minimum. LASSO, least absolute shrinkage and selection operator

**Fig. 2:**
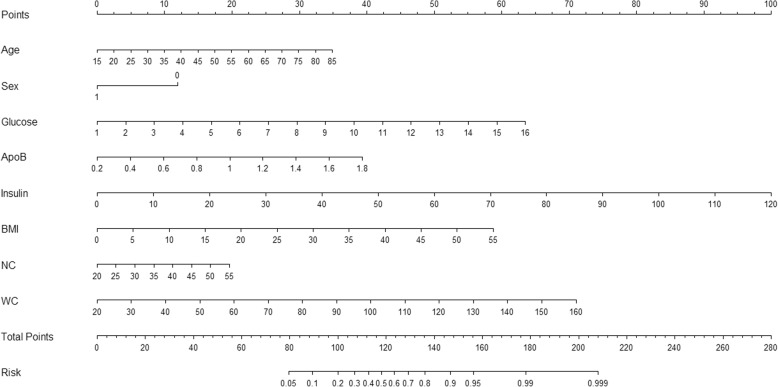
Our simple-to-use nomogram established for predicting OSA. The nomogram was developed in the training group by incorporating the following eight parameters: age (years), sex (0 = male; 1 = female), glucose (mmol/L), ApoB (g/L), insulin (μU/mL), BMI (kg/m^2^), NC (cm), and WC (cm). OSA, obstructive sleep apnea; ApoB, apolipoprotein B; NC, neck circumference; WC, waist circumference; BMI, body mass index

The LASSO method is suitable for regression of highly dimensional data, as it can be used to extract the most important predictive factors from a primary dataset. In this study, a risk score was calculated for each subject via a linear combination of factors that were weighted by their coefficients. Eighteen variables were reduced to eight potential predictors using the LASSO regression model. Then, a coefficient profile plot was produced (Fig. 1a). A cross-validated error plot of the LASSO regression model is shown in Fig. 1b. The most regularized and parsimonious model, with a cross-validated error within 1 standard error of the minimum, included eight variables. The path of the coefficients included in this model, with varying log-transformed lambda values, is shown in Fig. 1b. The model incorporated eight independent predictors (age, sex, glucose, ApoB, insulin, BMI, NC, and WC) and was developed as a simple-to-use nomogram (Fig. 2).

### Validation of the nomogram

**Fig. 3:**
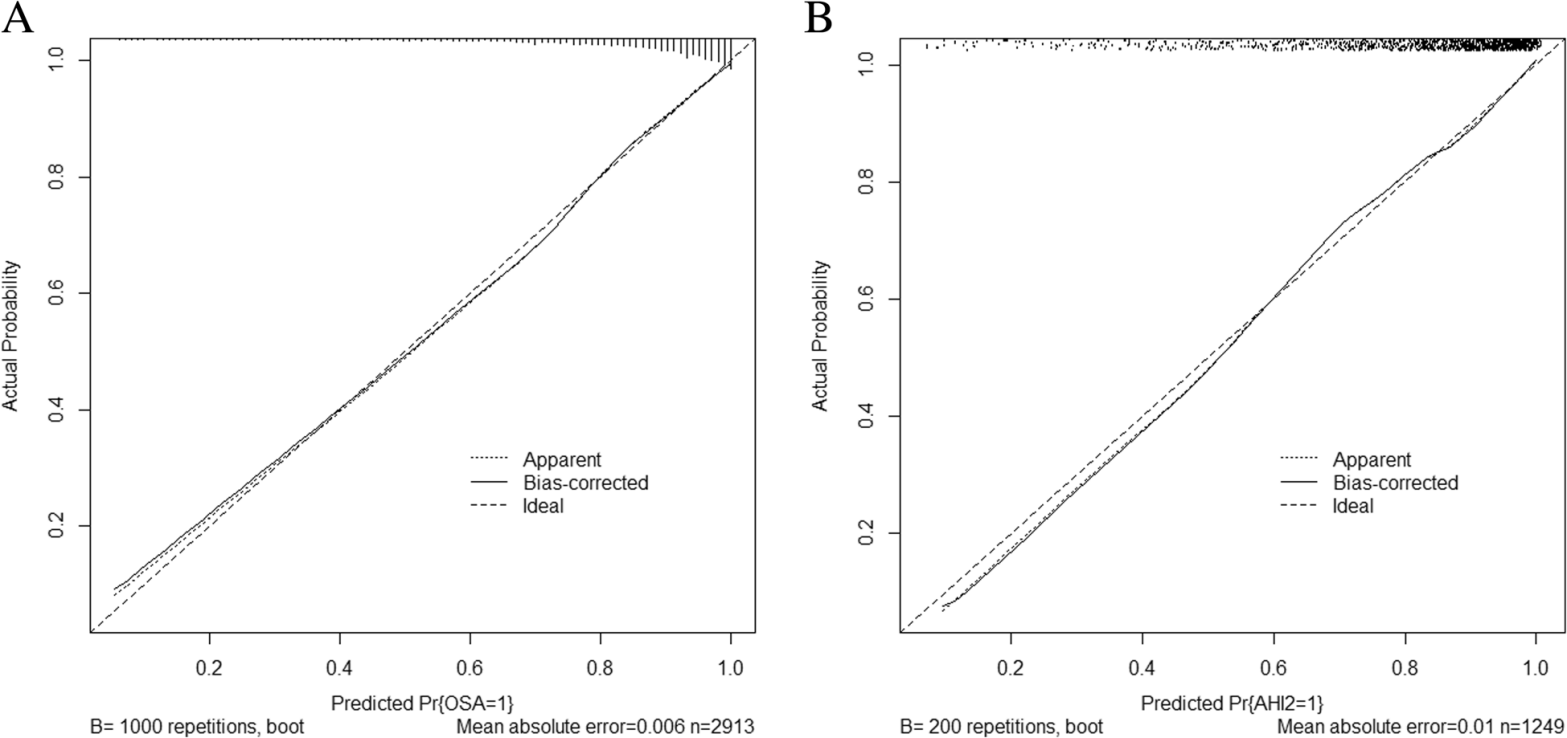
Calibration curves of the nomogram. **a** Calibration curve for the nomogram in the training group. **b** Calibration curve for the nomogram in the validation group

Validation of the nomogram was performed with a 1000 bootstrap analysis. In terms of the prediction of OSA, the C-index for the nomogram was 0.839 in the training group. In the validation group, the C-index was 0.820, which exceeded 0.7 and thus indicated that the model is suitable and sufficiently accurate for patients with OSA. The calibration plots demonstrated an excellent correlation between observed and predicted OSA in both the training (Fig. 3a) and validation groups (Fig. 3b).

### LASSO feature regression model

**Fig. 4:**
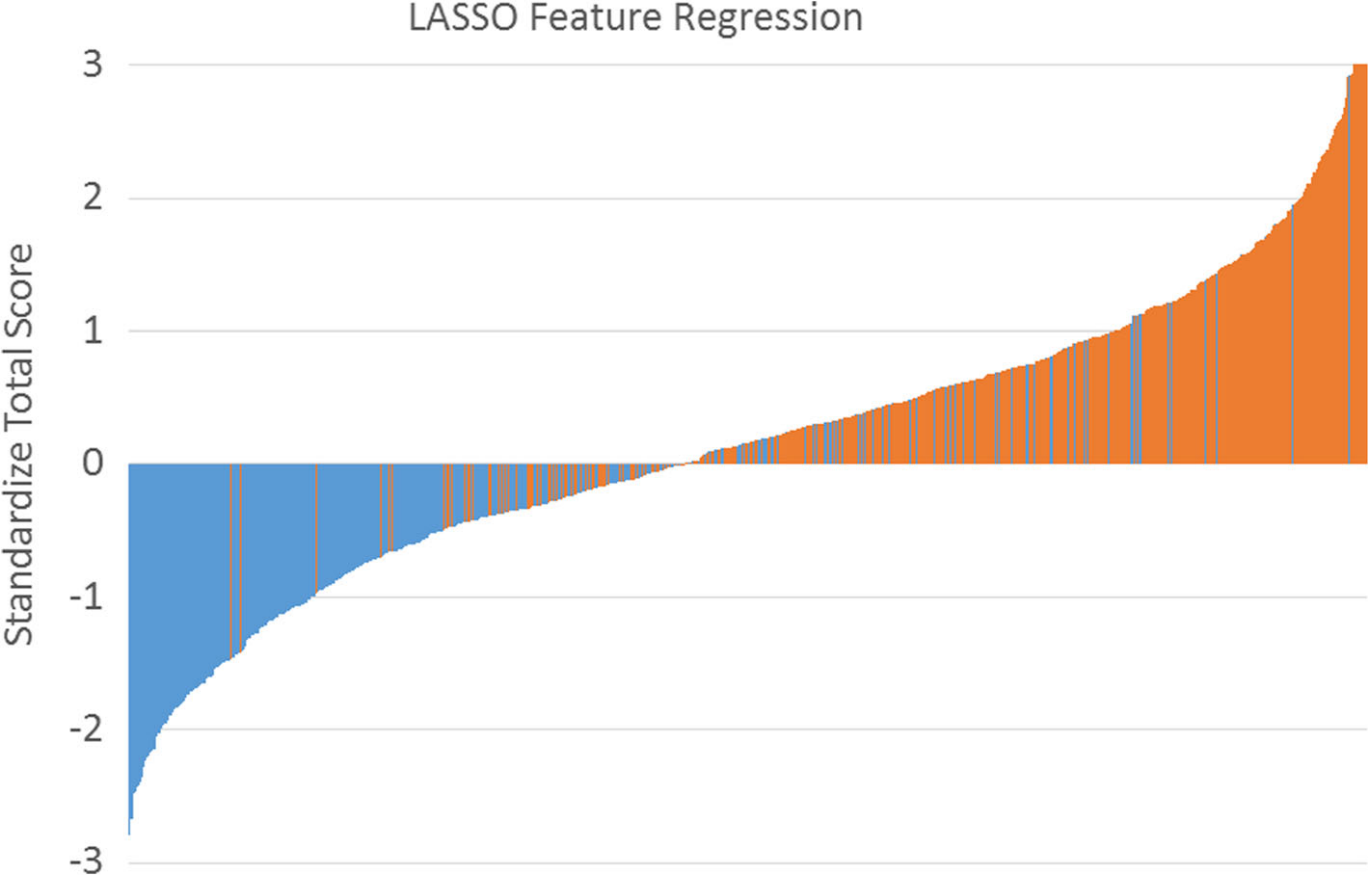
The LASSO feature regression. Standardized total score for each participant in the training group. Green bars represent scores for subjects without OSA, and red bars represent scores for those with OSA

We established a LASSO feature regression model to visualize the differences between OSA and non-OSA directly. The cutoff point to distinguish OSA from non-OSA was 137.2 (which was calculated from the nomogram). All patients had a nomogram score, which were standardized using the following formula: (nomogram score − 137.2)/standard deviation. The y-axis represents the calculated value; the x-axis represents each patient (green bars are non-OSA and red bars are OSA). In our study, we included more OSA participants than non-OSA participants; thus, too many subjects with OSA resulted in the accumulation of colored blocks. So we randomly selected 537 OSA and with 537 non-OSA patients together to establish the LASSO feature regression model (Fig. 4). We also used this nomogram to distinguish OSA from non-OSA, non-moderate to severe and non-severe OSA using AHI cutoffs of 5, 15 and 30 events/hour. The ROC curves showed that the optimum diagnostic cutoff point for the nomogram was relatively good (AUC = 0.84, AUC = 0.80, and AUC = 0.78 respectively, Table 2).

**Table 2:** The efficiency of nomogram for detecting OSA

	AUC	Sensitivity	Specificity	Positive likelihood ratio (+LR)	Negative likelihood ratio (−LR)	PPV	NPV
> 5	0.84 (0.83–0.86)	0.77 (0.76–0.79)	0.76 (0.72–0.80)	3.22 (3.10–3.40)	0.30 (0.30–0.40)	0.93 (0.92–0.94)	0.43 (0.41–0.46)
> 15	0.80 (0.79–0.82)	0.75 (0.730–0.77)	0.72 (0.69–0.75)	2.66 (2.50–2.80)	0.35 (0.30–0.40)	0.83 (0.82–0.85)	0.60 (0.58–0.62)
> 30	0.78 (0.76–0.79)	0.73 (0.71–0.76)	0.69 (0.66,0.71)	2.34 (2.20–2.50)	0.39 (0.30–0.40)	0.70 (0.68–0.71)	0.73 (0.71–0.74)

Data in parentheses are 95% CIs

*AUC* area under the curve, *PPV* positive predictive value, *NPV* negative predictive value

### An example for nomogram usage

To help new users understand how to proceed with this nomogram, we took one severe patients with OSA (AHI = 43.5 events per hour) for example (Table 3). This subject is male, 51 years old, with mean of glucose, ApoB, insulin, BMI, NC, and WC 5.26 mmol/L, 1.02 g/L, 14.21 μU/mL, 26.03 kg/m^2^, 40 cm, 95 cm, respectively. According to this nomogram, the total number of points for such a person is 158.5 (>cutoff of 137.2). This means this person referred to our sleep center, 96% risk to be OSA. He will be arranged to PSG monitoring earlier.

**Table 3:** an example of our simple-to-use nomogram usage

Characteristics	Person	
	Value	Points
age (years)	51	17.5
sex (0 = male; 1 = female)	0	12.5
glucose (mmol/L)	5.26	18
ApoB (g/L)	1.02	20
insulin (μU/mL)	14.21	12.5
BMI (kg/m^2^)	26.03	28
NC (cm)	40	12
WC (cm)	95	38
Total number of points^a^	(−)	158.5

Person: patients with severe OSA (AHI = 43.5 events per hour), male, 51 years old, with mean of glucose, ApoB, insulin, BMI, NC, and WC 5.26 mmol/L, 1.02 g/L, 14.21 μU/mL, 26.03 kg/m^2^, 40 cm, 95 cm, respectively

*OSA* obstructive sleep apnea, *ApoB* apolipoprotein B, *NC* neck circumference, *WC* waist circumference, *BMI* body mass index

^a^The sum of points attributable for each patient’s characteristic

## Discussion

In this study, we developed and validated an easy-to-use nomogram as a new approach to diagnose OSA in a large clinical sample. The nomogram incorporates eight items: age, sex, glucose, ApoB, insulin, BMI, NC, and WC. To our knowledge, this is the first study to establish an objective model, including common demographic, anthropometric, and biochemical variables, to distinguish OSA from non-OSA. The nomogram showed good accuracy and discrimination.

In total, 18 candidate variables were used for construction of the nomogram, which was reduced to eight potential predictors using the LASSO regression method. LASSO is suitable for analyzing large sets of clinical factors and avoids overfitting [20]. Our nomogram suggested that obesity and presence of a glucose metabolic disorder may be good predictors of OSA. Furthermore, the nomogram may serve as a useful tool for optimal identification of patients at high risk for OSA. Thus, therapeutic decisions will be better informed and the likelihood of early intervention for high risk patients would be increased, particularly in clinics lacking standard PSG equipment.

Luo M, et al. also established nomogram encompasses lots of subjective variables through an ordinal logistic regression procedure [21]. They found that the discrimination accuracies of this nomogram for non-OSA, moderate-severe OSA, and severe OSA were 83.8, 79.9, and 80.5%, respectively. Our study has a bit lower sensitivities, but with similar AUC (nearly 0.8). The biggest difference from the above-mentioned research is our study used objective parameters which could be avoiding bias caused by questionnaire recalled by patients. Besides, our study also had some advantages such as using LASSO regression for analyzing clinical factors, 10-fold subjects than previous study and had validation group. Other studies also used clinical nomograms to calculate AHI and even estimate median survival time/event-free survival [7, 22]. We will also extend the usage of nomograms in OSA in further prospective studies.

OSA remains underdiagnosed and undertreated in clinical settings, given the substantial burden that it confers. Thus, several questionnaire-based prediction tools for OSA have been developed and validated. For example, in a recent meta-analysis, the sensitivity (specificity) of the BQ, SBQ, STOP, and ESS for detecting mild OSA was 76% (59%), 88% (42%), 87% (42%), and 54% (65%), respectively; for moderate OSA, the corresponding values were 77% (44%), 90% (36%), 89% (32%), and 47% (62%); and for severe OSA they were 84% (38%), 93% (35%), 90% (28%), and 58% (60%) [12]. Although the SBQ seems to be more accurate than the other three questionnaires for screening OSA, its relatively low specificity limits its clinical utility [12]. One study assessed the ability of a simple, two-part questionnaire to predict OSA in a clinical setting; it showed high sensitivity (96.6%) but relatively low specificity (40.4%) [23]. However, the sample size (128 patients) in that study was relatively small. Shah et al. established a predictive model for sleep apnea that included age, BMI, snoring, and sex, and had a sensitivity of 0.77 and specificity of 0.75 [24]. However, a portable PSG rather than standard PSG was used in the Hispanic Community Health Study/Study of Latinos (HCHS/SOL) study, which may therefore have underestimated the severity of sleep apnea and also did not assess sleep stage, duration, or fragmentation (as noted by the authors [24]. The NoSAS score, a new screening tool that encompasses NC, BMI, snoring, age, and sex, was developed and validated in the HypnoLaus and EPISONO sleep cohort studies [25]. The NoSAS score seems marginally superior versus the BQ and ESS; however, its sensitivity and specificity are also low. Our objective nomogram seemed to perform better than traditional subjective questionnaires, however, this conclusion needs to be treated with caution because of had no validation in other ethnic groups.

Our study was performed according to previously described screening principles (WHO, 1968). We employed a rigorous methodology and an appropriate cross-sectional study design. Although our study had strengths, such as a large sample size, and all samples were evaluated by standard PSG, certain limitations should also be addressed. First, we only used demographic, anthropometric, and biochemical data to establish the nomogram; however, OSA is also affected by genetic factors, as evidenced by genome-wide association studies, and we did not consider genomic characteristics. Second, although our nomogram was developed in the context of a large sample size, and internal validation and external validation were performed, it has not been validated in different ethnic groups and populations. Third, the validation group was derived from the same institution as the training group, which makes it difficult to generalize the results to other populations. Therefore, we plan to externally validate our predictive model at other institutions. Fourth, the prevalence of OSA in our sleep center is higher than that in the general population; this high prevalence of OSA may have affected our evaluation of the predictive parameters by inflating their positive predictive value. Fifth, the assessment of biochemical variables raises concerns in term of money, time, and effort when compared with simple screening questionnaires. Lastly, the use of AASM 2007 scoring rules was not justified and could also be one of the potential limitations of our study.

## Conclusions

In conclusion, our simple-to-use nomogram, which incorporates demographic, anthropometric, and biochemical parameters, is considered to be a convenient tool for identifying undiagnosed OSA. This intuitive risk assessment tool may be useful for high-risk OSA subjects in clinical settings.

## Additional file

Additional file 1: Introduction of and how to perform LASSO regression method (described by Pripp AH, et el’s study). (DOCX 18 kb)

## Abbreviations

AHI: Apnea-hypopnea index
ApoA-I: Apolipoprotein A-I
ApoB: Apolipoprotein B
AUC: Area under the curve
BMI: Body mass index
ESS: Epworth Sleepiness
HC: Hip circumference
LASSO: Least absolute shrinkage and selection operator
Lp(a): Lipoprotein (a)
NC: Neck circumference
OSA: Obstructive sleep apnea
PSG: Polysomnography
ROC: Receiver operating characteristic
SBQ: STOP-Bang questionnaire
Scale BQ: Berlin questionnaire
WC: Waist circumference

## References

[1] Jordan AS, McSharry DG, Malhotra A (2014). Adult obstructive sleep apnoea. Lancet.

[2] Martinez-Garcia MA, Capote F, Campos-Rodriguez F, Lloberes P, Diaz de Atauri MJ, Somoza M, Masa JF, Gonzalez M, Sacristan L, Barbe F (2013). Effect of CPAP on blood pressure in patients with obstructive sleep apnea and resistant hypertension: the HIPARCO randomized clinical trial. JAMA.

[3] Tan X, van Egmond L, Chapman CD, Cedernaes J, Benedict C. Aiding sleep in type 2 diabetes: therapeutic considerations. Lancet Diabetes Endocrinol. 2018;6(1):60–8. 10.1016/S2213-8587(17)30233-4 28844889

[4] Xu H, Yi H, Guan J, Yin S (2014). Effect of continuous positive airway pressure on lipid profile in patients with obstructive sleep apnea syndrome: a meta-analysis of randomized controlled trials. Atherosclerosis.

[5] Leng Y, McEvoy CT, Allen IE, Yaffe K (2017). Association of sleep-disordered breathing with cognitive function and risk of cognitive impairment: a systematic review and meta-analysis. JAMA Neurol.

[6] Fu Y, Xia Y, Yi H, Xu H, Guan J, Yin S (2017). Meta-analysis of all-cause and cardiovascular mortality in obstructive sleep apnea with or without continuous positive airway pressure treatment. Sleep Breath.

[7] Kendzerska T, Gershon AS, Hawker G, Leung RS, Tomlinson G (2014). Obstructive sleep apnea and risk of cardiovascular events and all-cause mortality: a decade-long historical cohort study. PLoS Med.

[8] Young T, Palta M, Dempsey J, Skatrud J, Weber S, Badr S (1993). The occurrence of sleep-disordered breathing among middle-aged adults. N Engl J Med.

[9] Peppard PE, Young T, Barnet JH, Palta M, Hagen EW, Hla KM (2013). Increased prevalence of sleep-disordered breathing in adults. Am J Epidemiol.

[10] Heinzer R, Vat S, Marques-Vidal P, Marti-Soler H, Andries D, Tobback N, Mooser V, Preisig M, Malhotra A, Waeber G (2015). Prevalence of sleep-disordered breathing in the general population: the HypnoLaus study. Lancet Respir Med.

[11] Flemons WW, Douglas NJ, Kuna ST, Rodenstein DO, Wheatley J (2004). Access to diagnosis and treatment of patients with suspected sleep apnea. Am J Respir Crit Care Med.

[12] Chiu HY, Chen PY, Chuang LP, Chen NH, Tu YK, Hsieh YJ, Wang YC, Guilleminault C. Diagnostic accuracy of the Berlin questionnaire, STOP-BANG, STOP, and Epworth sleepiness scale in detecting obstructive sleep apnea: a bivariate meta-analysis. Sleep Med Rev. 2017;36:57–70. 10.1016/j.smrv.2016.10.004 27919588

[13] Bibbins-Domingo K, Grossman DC, Curry SJ, Davidson KW, Epling JW, Garcia FA, Herzstein J, Kemper AR, Krist AH, Force USPST (2017). Screening for obstructive sleep apnea in adults: US Preventive Services Task Force recommendation statement. JAMA.

[14] Xu H, Guan J, Yi H, Zou J, Meng L, Tang X, Zhu H, Yu D, Zhou H, Su K (2016). Elevated low-density lipoprotein cholesterol is independently associated with obstructive sleep apnea: evidence from a large-scale cross-sectional study. Sleep Breath.

[15] Bonora E, Targher G, Alberiche M, Bonadonna RC, Saggiani F, Zenere MB, Monauni T, Muggeo M (2000). Homeostasis model assessment closely mirrors the glucose clamp technique in the assessment of insulin sensitivity: studies in subjects with various degrees of glucose tolerance and insulin sensitivity. Diabetes Care.

[16] Iber C, Ancoli-Israel S, Chesson AL, Quan S. The AASM manual for the scoring of sleep and associated events: rules, terminology and technical specifications. Westchest IL Am Acad Sleep Med. 2007.

[17] Friedman J, Hastie T, Tibshirani R (2010). Regularization paths for generalized linear models via coordinate descent. J Stat Softw.

[18] Sauerbrei W, Royston P, Binder H (2007). Selection of important variables and determination of functional form for continuous predictors in multivariable model building. Stat Med.

[19] Wolbers M, Koller MT, Witteman JC, Steyerberg EW (2009). Prognostic models with competing risks: methods and application to coronary risk prediction. Epidemiology.

[20] Hepp T, Schmid M, Gefeller O, Waldmann E, Mayr A (2016). Approaches to regularized regression - a comparison between gradient boosting and the lasso. Methods Inf Med.

[21] Luo M, Zheng HY, Zhang Y, Feng Y, Li DQ, Li XL, Han JF, Li TP (2015). A nomogram for predicting the likelihood of obstructive sleep apnea to reduce the unnecessary polysomnography examinations. Chin Med J.

[22] Bucca C, Brussino L, Maule MM, Baldi I, Guida G, Culla B, Merletti F, Foresi A, Rolla G, Mutani R (2011). Clinical and functional prediction of moderate to severe obstructive sleep apnoea. Clin Respir J.

[23] Fenton ME, Heathcote K, Bryce R, Skomro R, Reid JK, Gjevre J, Cotton D (2014). The utility of the elbow sign in the diagnosis of OSA. Chest.

[24] Shah N, Hanna DB, Teng Y, Sotres-Alvarez D, Hall M, Loredo JS, Zee P, Kim M, Yaggi HK, Redline S (2016). Sex-specific prediction models for sleep apnea from the Hispanic Community Health Study/Study of Latinos. Chest.

[25] Marti-Soler H, Hirotsu C, Marques-Vidal P, Vollenweider P, Waeber G, Preisig M, Tafti M, Tufik SB, Bittencourt L, Tufik S (2016). The NoSAS score for screening of sleep-disordered breathing: a derivation and validation study. Lancet Respir Med.

