# Supplementary material for: Development and validation of a simple-to-use clinical nomogram for predicting obstructive sleep apnea
Source: BMC Pulm Med. 2019 Jan 18;19:18. doi: 10.1186/s12890-019-0782-1 (PMC6339352; doi:10.1186/s12890-019-0782-1)
Supplement: Supplementary file 1 — Introduction of and how to perform LASSO regression method (described by Pripp AH, et el’s study). (DOCX 18 kb) [file 12890_2019_782_MOESM1_ESM.docx]

**Supplementary Material and Data**

**Methods**

***Introduction of LASSO regression method***

LASSO regression is a statistical method that performs both factor selection and regularization. This implies mathematical procedures that tune and select the preferred level of model complexity to enhance the prediction accuracy, interpretability and generalization of the statistical model [1]. It is also used to make prediction models in a dataset with many and often inter-correlated independent variables in biological and medical research [2].

***How to perform LASSO regression method?***

The LASSO logistic regression was performed using the “glmnet” package in R (ver. 3.0.1; R Development Core Team, Vienna, Austria). The nomogram and calibration curve were plotted using the “rms” package.

***Mathematical foundations of LASSO regression method***

LASSO is a penalized method for restricting the residual sum of squares (deviance) and constraining the sum of the absolute values of the regression coefficients: For a binomial or continuous dependent variable, the outcome Y is either the original dependent variable Y (cf. linear regression) or Y = log [p/(1-p)], with p as the probability of the binary event (cf. logistic regression), respectively. A full regression model for k independent variables with coefficients β is:

Y = β0+β1X1 + β2X2 + β3X3 + ⋯ + βkXk (1)

The sum of absolute values of the coefficients is then estimated with the following restriction:


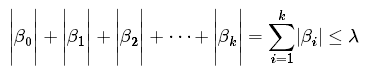
(2)

where λ is the “tuning” parameter. As λ approaches indefinite, it has no effect and the solutions are estimates for the full and unrestricted model. For smaller λ values, solutions are shrunken versions of the estimates, with many coefficients decreased to the null value. The selected value of λ was defined using cross-validation. The k independent variables are prior to fitting the model standardized to a mean of 0 and a standard deviation of 1. The solution of this fitted regression model is then presented with coefficients returned to the original scale, but typically with many coefficients decreased to the null value. Almost all description of the LASSO regression was derived from Pripp AH, et el’s study [3].

For more detail about LASSO regression, please see: https://en.wikipedia.org/wiki/Lasso (statistics).

**References:**

[1] Tibshirani R. Regression shrinkage and selection via the Lasso. J R Stat Soc Ser B-Methodol. 1996; 58(1):267–88.

[2] Tibshirani R. Regression shrinkage and selection via the lasso: A retrospective. Journal of the Royal Statistical Society Series B-Statistical Methodology. 2011;73: 273–82.

[3] Pripp AH, Stanišić M. Association between biomarkers and clinical characteristics in chronic subdural hematoma patients assessed with lasso regression. PLoS One. 2017;12(11):e0186838.
